# Oncocytic Features in Choroid Plexus Tumors: An Integrative Clinicopathological Study

**DOI:** 10.7759/cureus.85331

**Published:** 2025-06-04

**Authors:** Eliezer Villanueva-Castro, Marco Antonio Muñuzuri-Camacho, Martha Lilia Tena-Suck, Citlaltépetl Salinas Lara, Carlos Sánchez-Garibay

**Affiliations:** 1 Neurosurgery, National Institute of Neurology and Neurosurgery Manuel Velasco Suárez, Mexico City, MEX; 2 Neuropathology, National Institute of Neurology and Neurosurgery Manuel Velasco Suárez, Mexico City, MEX

**Keywords:** cellular senescence, choroid plexus tumors, immunohistochemistry, oncocytic changes, oncocytoma

## Abstract

Background: Choroid plexus tumors (CPTs) are rare epithelial brain tumors. Recent studies have found changes in the cells and the presence of amyloid, but these tiny details are still not well understood. Limited information is available regarding the biology of oncocytic changes (OCs) in CPTs.

Material and methods: This study included 42 primary and recurrent CPTs. Out of these, 28 cases (67%) were grade I choroid plexus papilloma (CPP), eight cases (19%) were grade II atypical choroid plexus papilloma (ACPP), and six cases (14%) were grade III choroid plexus carcinomas (CPCs), based on the World Health Organization (WHO) 2021 classification.

Results: We observed OCs in 10 (36%) CPP cases, three (38%) ACPP cases, and one (17%) CPC case (p = 0.643). We correlated these changes with mortality (p = 0.049), necrosis (p = 0.014), hemorrhage (p = 0.000), amyloid deposition, psammoma bodies, and brain invasion. OCs showed a positive reaction to fascin, CD1a, interleukin (IL)-2, IL-6, tumor necrosis factor-alpha (TNF-α), CD68, and hypoxia-inducible factor 1-alpha (HIF-1α), but the epithelial cells in CPP and CPC did not react. The detailed examination showed both normal and unusual mitochondria, fat droplets, thread-like materials, microtubules, amyloid buildup, and problems with cilia.

Conclusion: OCs in CPTs are rare and likely associated with inflammatory, ischemic, and hypoxic conditions. These changes might show dendritic cells (DCs) that help protect the blood-brain barrier, monitor the immune system, or support aging cells that promote flexibility and healing in tissues. Furthermore, we linked them to ciliary dysfunction. We need further studies to better understand this rare phenomenon in CPTs.

## Introduction

Choroid plexus tumors (CPTs) are rare intracranial neoplasms, accounting for approximately 0.4% to 0.6% of all brain tumors and 1% to 4% of pediatric brain tumors, with 10% to 20% of cases diagnosed within the first year of life [1]. According to the World Health Organization (WHO) classification [2], CPTs are primarily categorized into choroid plexus papilloma (CPP) and choroid plexus carcinoma (CPC).

In addition to these well-established types, CPTs occasionally exhibit uncommon histological features such as mucinous degeneration, melanization, tubular gland-like structures, connective tissue alterations, cartilage, bone or fat metaplasia, amyloid deposition, psammoma bodies, and oncocytic changes (OCs). Among these, OCs are particularly rare and have been reported only sporadically. Their biological significance and clinical implications in CPTs remain poorly understood [1].

OCs are characterized by abundant eosinophilic, granular cytoplasm due to the accumulation of altered mitochondria. This phenomenon, typically associated with chronic inflammation or cellular stress, can lead to the development of hyperplastic or neoplastic oncocytic nodules. In the context of CPTs, OCs have been proposed as a rare benign variant of CPP, although current evidence is limited [3].

The objective of this study is to comprehensively characterize OCs in CPTs through detailed histopathological evaluation, special staining techniques, and ultrastructural analysis using transmission electron microscopy (EM). Additionally, this study explores the clinical significance of these changes by investigating the expression of fascin and hypoxia-inducible factor 1-alpha (HIF-1α), novel immunohistochemical markers that may reflect underlying immune responses or hypoxia-driven mechanisms in these tumors. By integrating these approaches, we aim to provide new insights into the biological behavior and potential diagnostic value of oncocytic alterations in CPTs.

## Materials and methods

Data from 42 patients with primary and recurrent CPTs were collected from the electronic records of the Department of Neuropathology at the National Institute of Neurology and Neurosurgery Manuel Velasco Suárez in Mexico City, Mexico, covering the years 1990 to 2009. This cohort constituted the basis of the present study.

The clinical variables analyzed included age, gender, tumor location and size, symptom onset, recurrence, and mortality. More information was gathered, including the age when diagnosed, how much of the tumor was surgically removed, any follow-up treatments, and the patient's current health, to evaluate what the current tumor grading system means for future outcomes.

All tumor specimens were fixed in 10% formalin and embedded in paraffin. After being cut to a thickness of 5 µm, sections were stained using hematoxylin and eosin. The histopathological features examined included the growth of small blood vessels, certain immune cells, long-term inflammation, tissue hardening, changes in blood vessels, tissue death, bleeding, differences in cell nuclei, unusual cell shapes, specific cell changes, small calcified structures, infiltration into brain tissue, and scarring in the brain. We also assessed the mitotic index.

Immunohistochemistry was done using important antibodies like fascin, CD1a, interleukin (IL)-2, IL-6, IL-10, tumor necrosis factor-alpha (TNF-α), transforming necrosis factor-gamma (TNF-γ), CD68, HIF-1α, human leukocyte antigen-DR (HLA-DR), and Ki-67.

Tissue sections were deparaffinized in xylene and rehydrated in graded ethanol. Antigen retrieval was performed by heat-induced epitope retrieval using citrate buffer (pH 6.0) at 99°C for 20 minutes in a laboratory microwave. Sections were incubated with primary antibodies overnight at 4°C. Detection was carried out using a commercial kit according to the manufacturer’s instructions.

Immunostaining strength was rated from 0 to 3, and the type (membrane, cytoplasm, or nucleus) was noted. Based on how many cells tested positive, tumors were divided into four groups: negative, less than 30%, 30-60%, or more than 60% positive. The Ki-67 labeling index (MIB-1) was found by counting how many tumor cell nuclei were stained positively out of 1000 in the areas with the most staining.

To study the tiny details of the tissue, samples from paraffin blocks were treated with 3% glutaraldehyde and 2% osmium tetroxide, dried with stronger and stronger ethanol, and then put in epoxy resin (Araldite-Epon). Very thin slices (60-80 nm) were cut using a Reichert Ultracut microtome, stained with lead citrate, and examined with a Japan Electron Optics Laboratory (JEOL) transmission EM.

We used IBM SPSS Statistics for Windows, Version 20 (Released 2011; IBM Corp., Armonk, New York, United States) to perform all statistical analyses. Means, medians, and standard deviations were calculated to establish cutoff points. Categorical variables were compared using Fisher’s exact test. Continuous variables were analyzed using the Student’s t-test. A p-value <0.05 was considered statistically significant.

Ethical considerations

This study was conducted in accordance with institutional ethical standards. It was approved by the Research and Ethics Committee of the National Institute of Neurology and Neurosurgery Manuel Velasco Suárez, under protocol number 77/23. As a retrospective study based on archival clinical and histopathological data, informed consent was waived. All data were anonymized to protect patient confidentiality, and the study complied with national and international guidelines for research involving human subjects.

## Results

A total of 42 cases were included in this study. According to the 2021 WHO classification [2], 28 cases (67%) were identified as CPP (grade I); eight cases (19%) were atypical choroid plexus papilloma (ACPP, grade II); and six cases (14%) were CPC (grade III). Of the total patients, 37 (62%) were female and 23 (42%) were male.

Regarding tumor location, we found 35 tumors (58%) in the supratentorial location and 25 tumors (42%) in the infratentorial location. Specifically, 11 tumors (18%) were in the lateral ventricles, six (10%) in the third ventricle, and 25 (42%) in the fourth ventricle.

Patient age ranged from 17 to 67 years, with a mean of 28.63 ± 1.52 years. The mean age for female patients was 41.42 ± 1.47 years, and for male patients, 45.39 ± 1.77 years. Tumor size ranged from 24 mm to 65 mm, with a mean diameter of 43.07 ± 1.45 mm. The duration of symptoms prior to diagnosis ranged from 8 to 25 months, with a mean of 15.93 ± 0.69 months. Table 1 summarizes the clinical and pathological features of subtypes of CPTs.

**Table 1:** Clinical and anatomopathological characteristics of patients with choroid plexus tumors according to WHO 2021 classification The study provided a summary of the demographic, clinical, and pathological characteristics of patients diagnosed with CPP, ACPP, and CPC. Statistical comparisons between groups were performed using ANOVA and Fisher’s exact test. y: years; mm: millimeters; mo: months; CPP: choroid plexus papilloma; ACPP: atypical choroid plexus papilloma; CPC: choroid plexus carcinoma

Variable	CPP n = 28 (%)	ACPP n = 8 (%)	CPC n = 6 (%)	p-value
Age (years)	34	23.5	21	0.016
Sex				0.085
Female	20 (71)	7 (88)	2 (33)	
Male	8 (29)	1 (12)	4 (67)	
Tumor size (mm)	36	4	46	0.011
Time of symptoms presentation (months)	13	11.5	10	0.071
Location				0.222
Supratentorial	10 (36)	3 (38)	2 (33)	
Infratentorial	18 (64)	5 (63)	4 (67)	
Ventricular site				0.006
Lateral ventricle	7 (25)	2 (25)	2 (33)	
3rd ventricle	5 (18)	1 (12)	0	
4th ventricle	16 (57)	5 (63)	4 (67)	
Recurrence	8 (29)	5 (63)	6 (100)	0.002
Brain invasion	8 (29)	5 (63)	6 (100)	0.002
Follow-up (months)	24	16	10	0.007
Death	6 (21)	4 (40)	6 (100)	0.001

**Figure 1:**
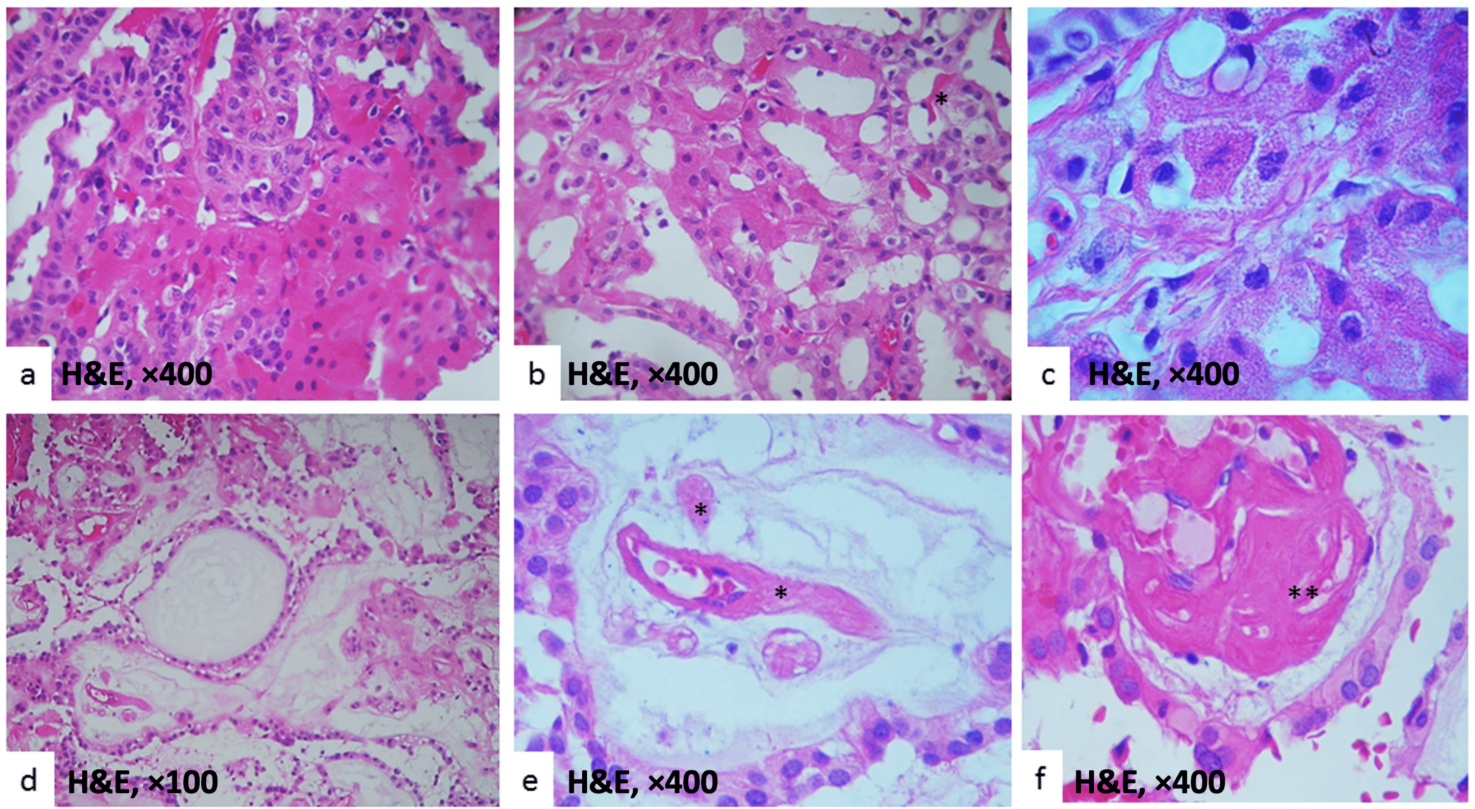
Histopathological features of CPP (a), (b), and (c) illustrate OC characterized by eosinophilic cytoplasm. (d) shows degenerative or hydropic changes in the papillae, indicating tissue expansion. (e) Amyloid fibrils appear as straight, non-branched structures. (f) Dense and scattered aggregates of amyloid fibrils are present between pericytes. The asterisk indicates amyloid fibrils. CPP: choroid plexus papilloma; H&E: hematoxylin and eosin

OCs were identified in 14 cases (33%) (Figures 1a-1c). Degenerative or hydropic alterations of the papillary structures were present in 18 cases (43%) (Figures 1d-1e). Psammoma bodies were observed in 11 cases (26%), and amyloid deposition was found in 14 cases (33%) (Figure 1f). Table 2 summarizes the main histological characteristics across subtypes of CPTs.

**Table 2:** Histological features observed in CPTs according to WHO 2021 classification Comparison of key histological features among CPP, ACPP, and CPC. We calculated the mitotic index as the median percentage of mitotic figures per high-power field. CPTs: choroid plexus tumors; CPP: choroid plexus papilloma; ACPP: atypical choroid plexus papilloma; CPC: choroid plexus carcinoma

Histological features	CPP n = 28 (%)	ACPP n = 8 (%)	CPC n = 6 (%)	p-value
Necrosis	2 (7)	4 (40)	6 (100)	0.300
Hemorrhage	8 (29)	6 (75)	6 (100)	0.393
Inflammation	6 (14)	7 (88)	8 (100)	0.000
Amyloid deposition	7 (25)	4 (50)	4 (67)	0.002
Psammoma bodies	9 (32)	5 (63)	2 (33)	0.008
Dystrophic calcification	9 (32)	7 (88)	2 (33)	0.005
Metaplastic changes	5 (18)	2 (25)	1 (17)	0.006
Oncocytic changes	10 (36)	3 (38)	1 (17)	0.643
Degenerative changes	12 (43)	3 (38)	2 (33)	0.222
Mitosis index (%)	1	4	21	0.035

**Figure 2:**
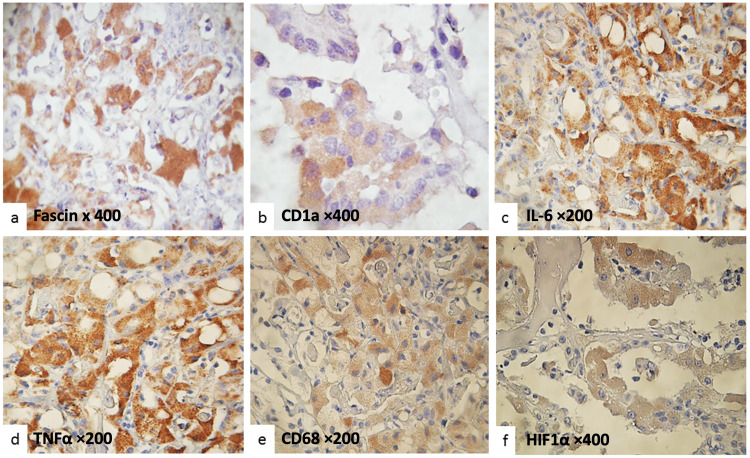
Immunohistochemical expression in oncocytic cells (a) Oncocytic cells exhibit diffuse cytoplasmic positivity for fascin. (b) CD1a expression is observed in epithelial cells, characterized by focal granular cytoplasmic staining. (c) Immunoreactivity for IL-6 is evident, with intense cytoplasmic staining in a solid pattern in tumor cells. (d) TNF-α expression is detected, showing diffuse cytoplasmic staining of moderate to strong intensity in cells with abundant clear cytoplasm. (e) CD68 positivity is present in neoplastic cells displaying a trabecular pattern. (f) HIF-1α expression is observed, with partial cytoplasmic staining in cells arranged in a papillary pattern. Normal epithelial cells show no staining. IL-6: interleukin-6; HIF-1α: hypoxia-inducible factor 1-alpha; TNF-α: tumor necrosis factor-alpha

Oncocytic cells tested positive for fascin, CD1a, IL-2, IL-6, TNF-α, CD68, and HIF-1α, while the other epithelial tumor cells did not show these markers (Figure 2). In contrast, the remaining epithelial tumor cells were negative for these markers. Table 3 presents the immunohistochemical results for subtypes of CPTs.

**Table 3:** Immunohistochemical analysis of choroid plexus tumors Immunohistochemical results for primary antibodies in CPTs subtypes. We calculated the Ki-67 labeling index as the median percentage of positively stained cells. The antibodies used are for fascin, CD1a (a marker for Langerhans cells), CD68 (a marker for macrophages), interleukins (IL-2, IL-6, IL-10), tumor necrosis factors (TNF-α, TNF-γ), HIF-1α (a factor that responds to low oxygen), HLA-DR (a type of protein important for the immune system), and Ki-67. Antibodies were obtained from Abcam (Ab), Santa Cruz Biotechnology (SC), and Dako (DAKO). CPT: choroid plexus tumors; IL: interleukin; TNF-α: tumor necrosis factor-alpha; TNF-γ: transforming necrosis factor-gamma; HIF-1α: hypoxia-inducible factor 1-alpha; HLA-DR: human leukocyte antigen-DR; CPP: choroid plexus papilloma; ACPP: atypical choroid plexus papilloma; CPC: choroid plexus carcinoma

Primary antibodies used	CPP n = 28 (%)	ACPP n = 8 (%)	CPC n = 6 (%)	p-value
Fascin-3 protein (tagged, ab162309)	4 (14)	5 (63)	6 (100)	0.300
CD1a (EP3622) (ab108309)	8 (29)	6 (74)	6 (100)	0.393
CD68 (DAKO, SPM130)	6 (21)	7 (88)	6 (100)	0.000
IL-2 ((C2-1-hIL2): sc-32295)	7 (25)	4 (50)	4 (67)	0.002
IL-6 (H-183)	4 (14)	5 (63)	2 (33)	0.008
IL-10 (sc-365858)	9 (32)	5 (63)	2 (33)	0.222
TNF-α (ab9635)	10 (36)	2 (25)	1 (17)	0.006
TNF-γ (TNF-γ, AbD Serotec, CC302)	10 (36)	3 (38)	1 (17)	0.643
HIF-1α (HIF-1α (28b))	14 (50)	3 (38)	3 (50)	0.222
HLA-DR	10 (36)	2 (25)	1 (17)	0.006
Ki67 (IW-1001, MIB-1, DAKO) (%)	4.5	9.5	16.5	0.002

**Figure 3:**
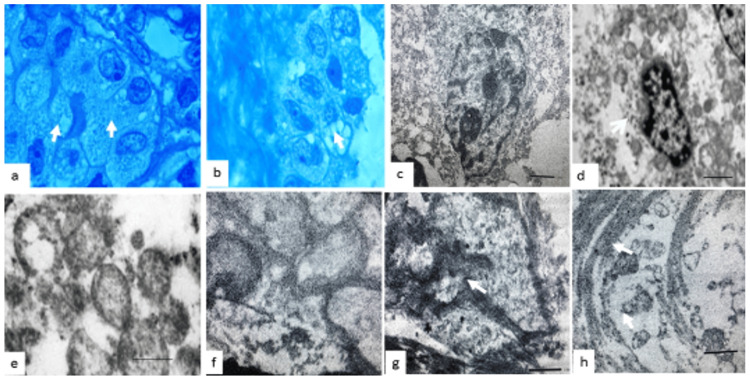
Ultrastructural characteristics of oncocytic cells (a) and (b) show ultrathin sections of epithelial cells, with oncocytic cells approximately twice the size of normal epithelial cells. (c) and (d) reveal ultrastructural features of oncocytes, including swollen mitochondria with abnormal cristae and large mitochondria with an empty or clear appearance. (e) and (f) provide close-up views of mitochondria with a clear appearance. (g) depicts interlocking cell processes, basement membrane disruption, and a fibrovascular core. (h) shows apical surface disruptions in epithelial cells, altered tight junctions, and clusters of cilia formations. Arrows indicate cilia clusters.

EM showed changes that might be due to the buildup of glycogen, lipids, hormones, and other materials, along with damage to cell structures (Figure 3). Examining the clear cells closely revealed that many mitochondria had lost their inner structure and folds, and the top part of the cell appeared empty. In CPP, we consistently observed both normal and abnormal cilia, along with an apical brush border, while in CPC, they were rarely identifiable. There were a few junctions (apical tight junctions) connecting the top and side parts of the cell membranes, and we also found glycogen granules.

Age and tumor size showed a linear association (p = 0.000). Furthermore, age was related to inflammatory response, brain invasion, degenerative changes, and mitotic index, with younger patients showing a higher mitotic index than older ones.

The size of the tumor was linked to necrosis (p = 0.004), inflammation (p = 0.003), invasion (p = 0.005), hydraulic changes (p = 0.020), mitotic features (p = 0.005), and age (p = 0.000). Nocytic changes were connected to death (p = 0.049), necrosis (p = 0.014), bleeding (p = 0.000), amyloid buildup (odds ratio: 1.471, 95% CI: 0.995-2.219), psammoma bodies (odds ratio: 1.350, 95% CI: 0.364-5.007), and invasion into the brain (p = 0.012, odds ratio: 1.333, 95% CI: 0.368-4.832). Hydropic changes (p = 0.012) were associated with gender (p = 0.036), with females exhibiting a significantly higher risk than males (odds ratio: 1.184, 95% CI: 0.291-4.825).

The Ki-67 labeling index (Ki67-LI) was correlated with patient age (p = 0.017), with older patients showing higher MIB-1 indexes. Tumors located in the fourth ventricle had a higher Ki67-LI compared to those in the lateral ventricles (p = 0.029), and these tumors tended to be larger (p = 0.019). Bigger tumors showed higher Ki67-LI, and there was a strong connection with the type of tissue, death, inflammation, signs of cell division, and amyloid buildup.

## Discussion

The terminology for oncocytic cells is varied. They have been referred to as Askenasi cells or oxyphilic cells [3,4] and are defined as cellular amplification characterized by abundant eosinophilic granular cytoplasm resulting from the accumulation of altered mitochondria [5,6], as well as the accumulation of diverse substances [4,7]. These cells have been observed in the central nervous system in tumors of the choroid plexus, meningiomas, and the pituitary gland [4]. Oncocytic tumors are predominantly found in endocrine tissues, including the thyroid, parathyroid, pituitary, adrenal cortex, and pancreas [5], and there is an oncocytic variant of renal cell and salivary gland carcinoma [5,6].

Oncocytic carcinomas usually present extra copies of 11q13.1-q13.2 and losses in 19p13. Given that oncocytic tumors in various locations have distinct clinical and biological traits, these chromosomal alterations may support mitochondrial accumulation and define a distinct molecular entity [8]. Mitochondrial mutations can lead to different inherited degenerative diseases, affecting all 13 genes of the oxidative phosphorylation (OXPHOS) system and resulting in RAS mutations [9].

Oncocytic transformation of the choroid plexus epithelium has been described [10], possibly as a compensatory mitochondrial hyperplasia due to damage or enzyme dysfunction [8,9]. Oncocytes are sometimes considered "burnt-out" cells, having lost their original specialization and increasing in number with age. Stefanko and Vuzevski and Giangaspero et al. were the first to describe the oncocytic variant of CPP, including its evolution from benign to malignant [3,11]. In our study and others, OC in CPTs appears to occur predominantly in adult patients and the fourth ventricle. While some studies suggest no clear biological behavior, others have linked OC with a higher risk of progression and recurrence after radiotherapy [8].

OC in CPTs can be subtle and go unnoticed. Jaiswal et al. [1] also observed clear cells in all CPTs, potentially indicating similarities between neoplastic and immature fetal choroid plexus tissue. Ultrastructural analysis has revealed approximately 10 nm amyloid fibrils along the basement membrane and vascular walls [3,4], although the relevance of these findings in CPTs remains unclear [7]. EM demonstrates key features such as increased oxidative activity, varied mitochondrial morphology, absence of dense granules, and lack of brush borders and basal infoldings. EM remains a valuable tool for identifying densely packed mitochondria, along with other findings like increased lysosomes, neurosecretory granules, and smooth endoplasmic reticulum [10-12].

In our study, OC was statistically associated with death, necrosis, hemorrhage, amyloid deposition, psammoma bodies, hydropic changes, and gender. Additionally, fascin and HIF-1α expression were evaluated due to their potential involvement in immune modulation and hypoxia. Fascin is an actin-bundling protein involved in migration, adhesion, and antigen presentation. While dendritic cells (DCs) and their cytokine profiles (e.g., IL-1, IL-6, IL-10, TNF-α) have been implicated in the choroid plexus microenvironment [13], our findings can only suggest potential immunological associations. For example, the presence of fascin may reflect interactions with DCs or local paracrine signaling, but we cannot infer direct mechanistic roles from our data.

Langerhans cells (LCs), a type of DC, exhibit distinct features such as Birbeck granules and CD1a expression, participating in non-classical antigen presentation [14]. Some markers (CD1a, MHC II) and cytokines (e.g., IL-10) influence antigen presentation and immune suppression [15]. However, the role of these cells in CPTs remains largely unexplored, and our findings should be interpreted as preliminary associations that warrant further investigation. Li et al. have proposed fascin and STAT3 pathways as contributors to invasiveness in glioma models [15], but such mechanisms remain speculative in CPTs.

Intraepithelial DCs in the choroid plexus may exist in a resting state, modulated by local signals [14,15]. Paracrine signaling, including cytokines and immune modulators, might contribute to tumor behavior, but these remain hypotheses not directly tested in our study [16].

EM findings in our series showed oncocytic cells with large mitochondria, mitochondrial remnants, lipid and glycogen deposits, and altered intercellular junctions. Structural ciliary defects and changes in the capillary basement membrane were also observed. These features may result from intracranial pressure changes and could impair cerebrospinal fluid regulation [17]. However, again, these are observational correlations without mechanistic confirmation.

Limitations of the study

This study has several limitations that should be considered when interpreting the findings. First, it is a retrospective series based on a relatively small sample size from a single institution, which may limit the generalizability of the results. Second, although immunohistochemical and ultrastructural analyses were performed, molecular profiling of the tumors was not included, which could provide further insights into the biological behavior of oncocytic variants. Third, the limited availability of follow-up data for certain cases, particularly CPCs, may introduce bias in survival and recurrence estimates. Lastly, due to the observational nature of the study, the associations observed between histological features, immunomarkers, and clinical outcomes should not be interpreted as causal. Future multicenter studies with molecular analysis and long-term follow-up are needed to validate these findings and clarify the clinical significance of OC in CPTs.

## Conclusions

OCs in CPTs are rare and often occur alongside features such as amyloid accumulation, psammoma bodies, and tissue swelling. These alterations may be associated with inflammatory processes, hypoxic conditions, tumor progression, and increased intracranial pressure. The presence of DCs suggests an activated immune response potentially serving as a protective mechanism at the blood-brain barrier. Ultrastructural findings indicate mitochondrial and ciliary abnormalities, which might reflect cellular aging or the impact of elevated intracranial pressure.

Importantly, our findings are observational and do not establish causal mechanisms. Further molecular and functional studies are necessary to elucidate the precise biological significance of these changes and their implications for CPT pathophysiology. This study provides a foundation for future research aimed at clarifying the clinical relevance of OCs in CPTs.
